# Engineered Bacteriophage T7 as a Potent Anticancer Agent *in vivo*

**DOI:** 10.3389/fmicb.2020.491001

**Published:** 2020-09-24

**Authors:** Yoon Jung Hwang, Heejoon Myung

**Affiliations:** ^1^ Department of Bioscience and Biotechnology, Hankuk University of Foreign Studies, Yong-In, South Korea; ^2^ Bacteriophage Bank of Korea, Hankuk University of Foreign Studies, Yong-In, South Korea; ^3^ LyseNTech, Yong-In, South Korea

**Keywords:** engineered phage T7, oncolytic virus, phage display, granulocyte macrophage-colony stimulating factor, anticancer

## Abstract

Oncolytic viruses (OVs) induce antitumor effect by both direct lysis of target cells and eliciting immunogenic response to the virus and ultimately to the target cells. These viruses are usually natural human pathogens. Bacteriophages are natural pathogens of bacteria that do not infect human and have greater advantages in safety, manipulation, and production over human viruses. We constructed an engineered bacteriophage T7 displaying a peptide, which targets murine melanoma cells and harbors a mammalian expression cassette of the cytokine granulocyte macrophage-colony stimulating factor (GM-CSF) in viral genomic DNA. The engineered phage was successfully transduced to B16F10 melanoma cells both *in vitro* and *in vivo*. GM-CSF was expressed from the transduced phage DNA. All mice treated with the phage intravenously survived for 25 days until the end of experiment, while only 40% of those not treated survived. During the 16 days of phage treatment, phage T7 displaying homing peptide and expressing GM-CSF inhibited tumor growth by 72% compared to the untreated control. Serum cytokine levels of IL-1α, TNF-α, and GM-CSF were seen to increase during the treatment. Immunohistochemical analysis of tumor tissue revealed infiltration by macrophages, dendritic cells (DCs), and CD8^+^ T cells. Migration of murine macrophages to bacteriophages was also observed in *in vitro* transwell assays in both time- and dose-dependent manners. Taken together, the recombinant bacteriophage T7 efficiently inhibited tumor growth by changing the tumor microenvironment and recruiting anti-tumor immune cells.

## Introduction

Oncolytic viruses (OV) are viruses, which are known to attack and lyse cancer cells (Raja et al., 2018; Reale et al., 2019). They destroy tumor mass by infecting and multiplying within the mass. In addition to viral lysis, the tumor mass, due to the presence of immunogenic viruses, is subject to attack by the immune system. Partial remission of tumor mass when patients contracted viral diseases was observed by physicians as early as the beginning of twentieth century (Kelly and Russell, 2007). An HSV-1 based oncolytic virus (T-VEC) has already been approved by the FDA and is currently in clinical use (Puzanov et al., 2016). Attenuated human pathogens, which have been tested as potential OVs, include the adenovirus (Shaw and Suzuki, 2016), the vaccinia virus (Haddad, 2017), the measles virus (Aref et al., 2016), the mumps virus (Ammayappan et al., 2016), and the influenza virus (Pizzuto et al., 2016). Other viruses known to be poor human pathogens, which have been tested as OVs, include the Newcastle disease virus (Tayeb et al., 2015) and the vesicular stomatitis virus (Bishnoi et al., 2018). Potential OVs are often not powerful enough for solid tumors and safety has not always been matched with efficacy, as OVs are known to exhibit a certain range of toxic effects (Raja et al., 2018; Reale et al., 2019). In addition, live viruses have been shown to be transferable from the primary treatment patient to healthcare workers and people inhabiting the same household (Robilotti et al., 2019).

Bacteriophages are viruses naturally infecting bacteria. Since their discovery independently by Frederick Twort and Felix d’Herelle in early twentieth century, phages have mainly served as antibacterial agents and for the exploration of the basic mechanisms of life at the molecular level. In 1940, accumulation of phages in cancer tissue and the inhibition of tumor growth were observed, and in 1958, the binding of phages to cancer cells was seen both *in vitro* and *in vivo* (Budynek et al., 2010). Phage T4 and its substrain HAP1 were shown to bind melanoma cells and inhibit lung metastasis in a murine model (Dabrowska et al., 2004). Interaction between lysine-glycine-aspartic acid (KGD) motif on phage capsid protein and the β3 integrin receptor on cell surfaces was hypothesized as being responsible for the activity. The same group of researchers showed that dendritic cells (DCs) primed with phage T4 and tumor antigens initiated differentiation accompanied by exhibiting an enhanced ability to prime T cells (Pajtasz-Piasecka et al., 2008). Application peritumorally of primed DCs retarded tumor growth in a mouse colon cancer model. Eriksson et al. reported that peritumoral injection of M13 phages displaying peptide receptors or Fab fragments in mouse melanoma cells led to delayed tumor growth and increased survival of tumor bearing mice (Eriksson et al., 2007). Later, by investigating the tumor microenvironment, the same group of researchers showed that tumor destruction was caused by the activation of tumor-associated macrophages (Eriksson et al., 2009). Hybrid adeno-associated virus/phage (AAVP), a filamentous phage capsid packaging the cis-acting AAV genomic element, was developed to facilitate targeted gene delivery without natural cell tropism (Hajitou et al., 2006). Arginine-glycine-aspartic acid (RGD) peptide-displaying AAVP expressing TNF-α with radiation therapy inhibited melanoma by modifying the tumor microenvironment in a mice model (Quinn et al., 2017).

In this study, we developed phage T7 as an oncolytic phage (OP). It displayed peptides targeting mouse melanoma cells and at the same time harbored a mammalian expression cassette for the cytokine granulocyte macrophage-colony stimulating factor (GM-CSF). We tested its anti-tumor efficacy *in vivo*.

## Materials and Methods

### Bacteriophage and Bacteria

The homing peptide used was pep42 (CTVALPGGYVRVC) targeting grp78 on cancer cells (Kim et al., 2006). Both strands of oligonucleotides encoding the peptide were synthesized (Bioneer, Korea) and annealed and ligated between EcoRI and HindIII sites in multiple cloning sites of T7 Select 10-3 cloning kits (Novagen, Canada). The resulting T7 Select vector was used for electroporation into *Escherichia coli* BLT5403 (Novagen, Canada) to produce peptide-displaying phages. Additionally, a cassette expressing GM-CSF under CMV promoter was synthesized (Bioneer, Korea) and used to clone into the above described T7 genomic DNA (GenBank accession number V01146.1), which was cut with the restriction enzyme PacI at 27,223~27,230 basepair. The synthesized cassette consisted of CMV promoter, KOZAK sequence, the ORF encoding murine GM-CSF (Gene ID 12981), and BGH polyA signal.

The recombinant phage was used to infect freshly cultured *E. coli* BLT5403 in a 500 ml culture media at the multiplicity of infection (MOI) of 0.1. The mixture was incubated at room temperature for phage adsorption for 1 h followed by shaking incubation at 37°C for 3 h. Chloroform was added to the culture at a final concentration of 5% (v/v) for complete lysis of bacteria and the culture was then incubated with shaking for 15 more min. NaCl was subsequently added at a final concentration of 6% (weight/volume) and the culture was incubated at 4°C for 1 h. To remove any remaining bacterial cells or debris, the mixture was subjected to centrifugation at 11,000 × *g* for 10 min. The supernatant was recovered and PEG8000 was added at a final concentration of 10% (weight/volume). The mixture was again subjected to centrifugation at 11,000 × *g* for 10 min. Supernatant was discarded and the pellet was resuspended in 1 ml of SM buffer (50 mM Tris-HCl pH7.5, 100 mM NaCl, and 8 mM MgSO_4_). One milliliter of chloroform was added and the mixture was rigorously vortexed and subjected to a centrifugation at 2,000 × *g* for 15 min. The upper phase was recovered and subjected to an ultracentrifugation. Three milliliter of 40% glycerol was poured into an empty tube followed by the slow addition of 5% glycerol. The upper phase containing phages was then added to the tube and the remaining space was filled with SM buffer. The tube was centrifuged at 270,000 × *g* for 1 hour. Supernatant was discarded and the pellet containing phages was recovered by resuspension in 1 ml of SM buffer.

### Removal of Endotoxins

The method was described previously (Branston et al., 2015). Triton X-114 was added to the phage sample at a final concentration of 1% (v/v) and the mixture was rigorously vortexed. After incubation on ice for 5 min, the mixture was rigorously vortexed and subjected to centrifugation at 15,000 × *g*, 37°C for 1 min. The upper phase was recovered and used for phage experiments *in vitro* and *in vivo*.

### Cancer Cell Line and Culture Conditions

The B16F10 mouse melanoma cell line (KCLB80008) was obtained from the Korean Cell Line Bank at Seoul National University. Cells were grown in Dulbecco’s Modified Eagle’s Medium (DMEM, Thermo Fisher Scientific, USA) supplemented with fetal bovine serum (FBS, CELLect, USA) at a final concentration of 10% (v/v) and penicillin/streptomycin (Sigma Aldrich, USA) at a final concentration of 1% (v/v).

### Confocal Microscopy Analysis of Phage Transduction

For staining bacteriophage T7 after transduction, 1 × 10^5^ B16F10 cells were seeded in a six-well plate with coverslip and grown in a CO_2_ incubator. Media was discarded after 24 h and 1 × 10^7^ PFU of phages in SM buffer were added to each well and incubated for 30 min. Unbound phages were washed out and the cells were fixed with cold acetone. Blocking solution (1% bovine serum albumin in PBS) was added and the mixture was incubated for 30 min. Cells were then treated with 1:500 diluted anti-T7 tag antibody (ab9138, Abcam, USA) for 1 h followed by washing with PBS three times. Secondary antibody (1:1000 diluted anti-goat antibody, ab6881 Abcam, USA) was added and the mixture was incubated for 1 h followed by washing with PBS three times. The nucleus was stained with 4', 6-diamidino-2-phenylindole (DAPI) for 5 min. A laser confocal microscope (LSM 700, Carl Zeiss, Germany) was used for observations. For masking the receptor grp78 prior to phage transduction, 1:100 diluted anti-grp78 antibody (ab21685, Abcam) was added to the cell culture and incubated for 1 h followed by washing with PBS. Visualization was performed by adding an Alexa594-labeled secondary antibody (anti-grp78 rabbit antibody, ab150064, Abcam). For the labeling of phage DNA, BrdU (Thermo Fisher Scientific, USA) was added at a final concentration of 300 μM at the time of phage infection to the bacterial culture and the resulting progeny phages were collected.

### Cytotoxicity Assay *in vitro*

A total of 3 × 10^4^ B16F10 cells were seeded in a 96-well plate and incubated overnight. Bacteriophages were added to the well at multiplicities of infection (MOI) of 10 or 100 and the mixture was incubated for 24 h followed by 3-(4,5-dimethylthiazol-2-yl)-2,5-diphenyltetrazolium bromide (MTT) assay (Cell Viability Assay Kit, Dong-In LS, Korea) in accordance with the manufacturer’s instructions.

### Expression of GM-CSF From Transduced Phage *in vitro*

A total of 1 × 10^6^ B16F10 cells were seeded in a six-well plate and incubated overnight. Engineered bacteriophage T7 displaying homing peptide (pep42) and expressing GM-CSF was added to the culture at an MOI of 100 and cells were incubated for 3 days. Cells were harvested and lysed with cell extraction buffer (50 mM Tris-HCl, pH8.0, 150 mM NaCl, 0.1% Triton X-100, 0.5% sodium dodecyl sulfate, 1 mM sodium orthovanadate, and 1 mM NaF) and the lysate was subjected to an SDS-PAGE analysis. Expression was confirmed using the anti-GM-CSF antibody (ab9741, Abcam, USA) and the anti-rabbit secondary antibody (ab6717, Abcam, USA) in a western blot analysis. The same lysate was used for the extraction of total RNA using the TRleasy Total RNA Ultrapurification kit (RBC, Taiwan). Fifty milliliter of extracted RNA was treated with 1 unit of DNase I (Solgent, Korea) at room temperature for 15 min for complete removal of DNA as described (Rio et al., 2010). The mixture was cleaned up through a column included in the above RNA isolation kit. Five microgram of total RNA was mixed with oligo (dT)_18_ primer and 1,000 U of reverse transcriptase (DyneBio, Korea). The mixture was annealed at 42°C for 5 min followed by incubation at 50°C for 60 min to allow for enzyme reaction. The reaction was then stopped by incubation at 70°C for 15 min. Real-time PCR (RT-PCR) was performed with the resulting cDNA, primer, and SYBR Green qPCR 2X PreMix (DyneBio, Korea). The primer sequence used was forward: 5'GGCCTTGGAAGCATGTAGAG3' and reverse: 5'CCGTAGACCCTGCTCGAATA3'. As a control, the extracted RNA with DNase I treatment was subjected to a PCR reaction to detect any remaining DNA.

### Animal Experiments

All animal studies were approved by, and complied with, the regulations and guidelines of the Ethical Committee for Animal Experiments of Hankuk University of Foreign Studies (approval number HUFS-2017-0002). Six-week-old female Balb/C mice were obtained for the experiments (Young Bio, Korea). For tumor size measurement, 30 mice were divided into six groups. *In vitro* cultured 5 × 10^6^ B16F10 cells were subcutaneously injected into the right flank of each mouse. Tumor mass was allowed to grow for 1 week until its diameter reached ca. 5 mm. Treatment started 1 week post melanoma cell graft. Group 1 contained control mice with SM buffer treatment. Group 2 mice were treated with 1 × 10^9^ PFU of wild type bacteriophage T7 every day for 10 days. Group 3 mice were treated with 1 × 10^9^ PFU of pep42-displaying bacteriophage T7 every day for 10 days. Group 4 mice were treated with 1 × 10^9^ PFU of pep42-displaying bacteriophage T7 harboring expression cassette of GM-CSF every day for 10 days. Group 5 mice were treated with 1 × 10^9^ PFU of pep42-displaying bacteriophage T7 and 1 ng of GM-CSF (catalogue number Z03300, GenScript, USA) every day for 10 days (Sun et al., 2018). Group 6 mice were treated with 1 ng of GM-CSF every day for 10 days. All treatments were injected intravenously (IV) in the tail vein. Tumor volume was measured during the treatment period. After 10 days of treatment, the mice were sacrificed and tumor mass was removed for immunohistological analysis. For serum cytokine analysis, 400 μl of orbital blood collection was performed for each mouse. For survival observations, 30 mice were divided into six groups as above and survival was monitored for 25 days. When tumor mass exceeded 10% of total body weight, the mouse was euthanized. The survival graph was plotted in accordance with Kaplan-Meier plot and drawn with Prism, GraphPad Software.

### *In vivo* Imaging

*In vivo* imaging was described previously (Kelly et al., 2006). Briefly, 1 × 10^9^ PFU of pep42-displaying bacteriophage T7 or wild type bacteriophage T7 was fluorescently labeled with 0.25 mg/ml of fluorochrome-hydroxyl-succinate ester (cy5.5) in a dark room at room temperature for 1 h. Cy5.5-labeled phages were injected in tail veins of Balb/C mice bearing B16F10 grafted tumor mass and *in vivo* live imaging was performed using an FMT 2500-LX imager (Institute Pasteur Korea) after 2 h.

### Cytokine ELISA

Serum was obtained from mouse blood by centrifugation at 1000 × *g* for 15 min. Mouse cytokines IL-1α, TNF-α, and GM-CSF were measured using Multi-Analite ELISArray Kits (Qiagen, Germany) in accordance with the manufacturer’s instruction. The assay was performed in triplicate.

### Immunohistological Analysis

Tumor-bearing mice treated with various phages and/or cytokine were sacrificed and tumor masses were removed. These were then fixed in 10% formalin and hematoxylin-eosin (HE) staining and immunohistochemistry (IHC) were performed (LogOne Bio Convergence Research Foundation, Seoul, Korea).

### Statistical Analysis

All experiments were performed in triplicate and statistical significance was obtained using one way ANOVA followed by Tukey’s test (Prism, GraphPad Software). *p* < 0.05 was considered as statistically significant. Kaplan-Meier analyses were used and the log-rank Mantel-Cox test was employed to determine any difference between the survival curves of the groups. *p* < 0.05 was considered as statistically significant.

### Transwell Migration Assay

Twelve millimeter transwell with 3.0 μm pore (corning transwell polyester membrane cell culture inserts, CLS3462) was used. A total of 1 × 10^5^ B16F10 cells were seeded in the lower chamber and incubated at 37°C for 24 h. A total of 1 × 10^7^ PFU of phage T7 displaying pep42 harboring expression cassette of GM-CSF was added to the confluently grown cells and incubated for 24 h. Then, 1 × 10^5^ Raw 264.7 cells were loaded in the upper chamber and incubated at 37°C for 2, 6, or 24 h. Media were discarded and migrated cells on membrane surfaces were fixed with 1 ml of 70% ethanol at room temperature for 5 min followed by drying for 15 min. Fixed cells were stained with 0.2% crystal violet at room temperature for 5 min followed by washing with distilled water three times. Cells were then observed under a light microscope.

## Results

**Figure 1:**
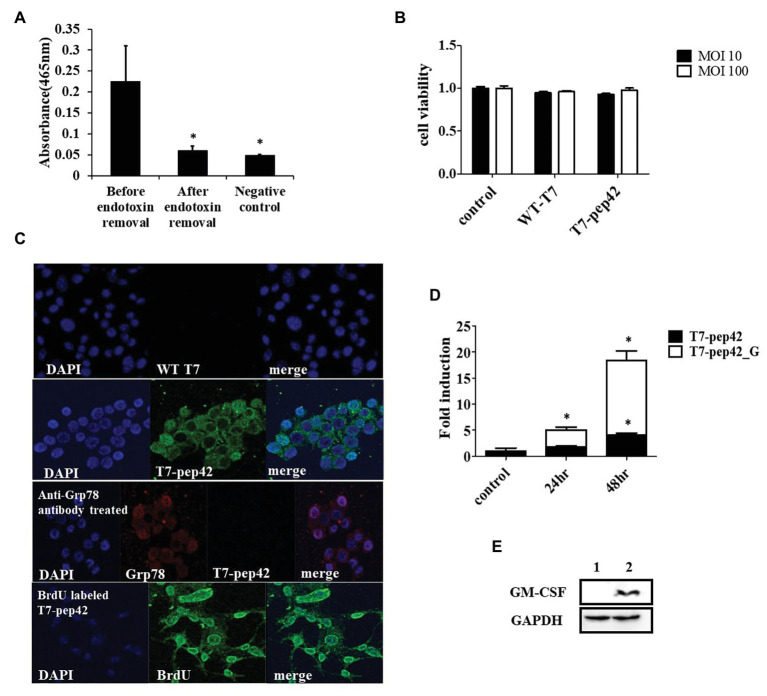
Phage preparation, homing, and expression of granulocyte macrophage-colony stimulating factor (GM-CSF) *in vitro*. **(A)** Removal of endotoxin after phage preparation. SM buffer was used as a control. **(B)** Cell viability assay of B16F10 cells after exposure to bacteriophages. Two different concentrations (multiplicity of infection of either 10 or 100) of native T7 or engineered T7 were added to the culture and incubated for 24 h before MTT assay was performed. Control was treated with SM buffer. **(C)** Homing and internalization of phage particle, and nuclear localization of phage DNA. First and second row: wild type phage T7 (WT T7) or T7 displaying the homing peptide (T7-pep42) was added to *in vitro* cultured B16F10 melanoma cells and binding was observed under a fluorescent laser scanning confocal microscope. The nucleus was stained with DAPI (blue) and the phage particle was stained with anti-T7 antibody (green). Third row: B16F10 cells were first treated with anti-Grp78 antibody (red) to mask the receptor for pep42. Then T7-pep42 (green) was added and binding was observed. Fourth row: T7-pep42 was produced in the presence of BrdU (green) to label the genomic DNA and added to cultured B16F10 cells. Internalized phage DNA to DAPI (blue) stained nucleus is shown. **(D)** Real-time PCR (RT-PCR) analysis of mRNA encoding GM-CSF from T7-pep42 or T7-pep42_G transduced B16F10 cells. Relative amounts of mRNA encoding GM-CSF from cells treated with T7-pep42 or T7-pep42_G are shown in black or white bars, respectively. Control was treated with SM buffer. T7-pep42, phage T7 displaying pep42 and T7-pep42_G, phage T7 displaying pep42 and expressing GM-CSF. **(E)** Western blot analysis of GM-CSF from T7-pep42_G transduced B16F10 cells (lane 2) and empty cell (lane 1). GAPDH was used as an internal control. For statistical analysis, one way ANOVA was performed and then Tukey’s test was conducted. ^*^Above each vertical bar indicates statistical significance of each test to the control. ^*^Above each horizontal bar indicates statistical significance of each test between corresponding pairs.

The engineered bacteriophage T7 displaying homing peptide (pep42) and harboring a mammalian expression cassette of murine GM-CSF was produced and the phage solution was nearly completely cleared (90%) of endotoxins (Figure 1A). We first tested if this preparation exerted any toxicity *in vitro*. An MTT assay was performed with the results indicating that neither wild type T7 nor its engineered version had a significant effect on the viability of murine melanoma cells (Figure 1B). Next, we verified whether the engineered phage homed into B16F10 cells *in vitro*. Both wild type T7 and its engineered version were added to cultures of murine melanoma cells and stained with anti-T7 antibody followed by observation under laser scanning confocal fluorescent microscope (Figure 1C). As anticipated, wild type T7 was washed out while engineered T7 displaying pep42 remained attached to melanoma cells. Grp78 is known to be the receptor for pep42 (Kim et al., 2006). When the melanoma cells were treated with anti-Grp78 antibody prior to the addition of phages, phage T7 displaying pep42 could not bind to the cells. We then grew phage T7 in the presence of BrdU to label the phage genomic DNA and used the phage for transduction. We observed localization of BrdU labeled DNA to the nucleus, suggesting that phage genomic DNA enters the nuclei of melanoma cells. Since the phage genomic DNA harboring the expression cassette of GM-CSF was localized to nuclei, we expected production of cytokine GM-CSF from the transduced culture of melanoma cells. We could observe the transcription of cassette in a time-dependent manner using RT-PCR analysis (Figure 1D). Expression of GM-CSF was also observed by western blot (Figure 1E).

**Figure 2:**
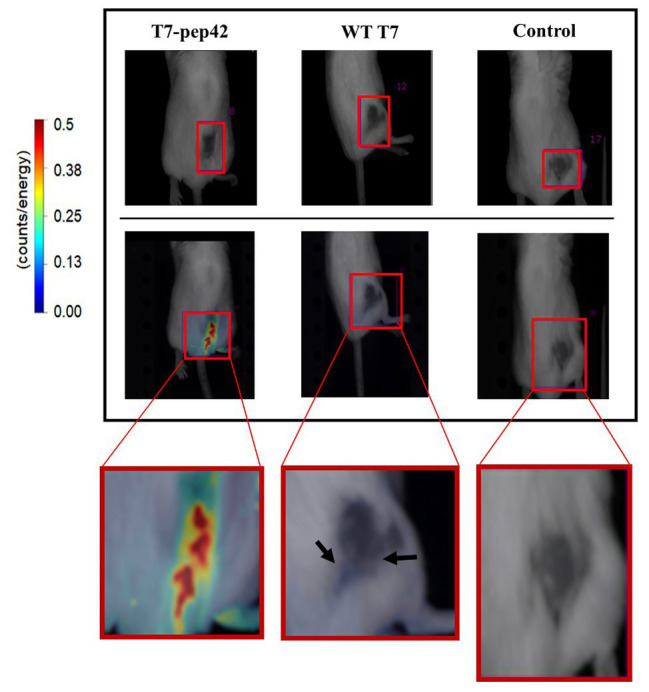
*In vivo* imaging of intravenously administered phages. cy5.5-labeled phages were injected in tail veins of Balb/C mice bearing B16F10 grafted tumor mass and *in vivo* live imaging was performed at 7 days post graft. The tumor mass is indicated in each red box and fluorescence was measured as counts/energy and the intensity is shown in different colors. **Upper panel**: light field observation. **Lower panel**: fluorescence observation. Boxes at the bottom show enlarged picture of the areas of interest for an easier comparison. Note the bluish area indicating a low amount of phage accumulation pointed by arrows in the middle box.

As homing of the engineered phage was seen *in vitro*, we next investigated whether it homed into targets *in vivo*. After grafting of *in vitro* grown B16F10 cells in the mice and after their tumor masses had built up, either wild type phage T7 or the engineered version was given to the mice intravenously and *in vivo* live imaging was performed (Figure 2). Fluorescently labeled phage T7 displaying pep42 localized to tumor masses four times more than wild type T7. The location of wild type T7 looked peritumoral, rather than intratumoral, while the location of the majority of the engineered T7 looked intratumoral.

**Figure 3:**
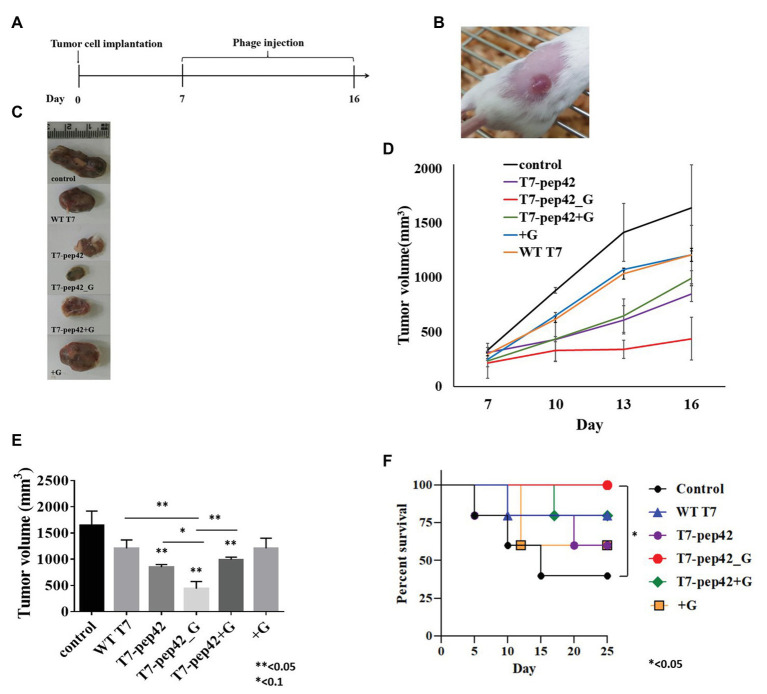
Animal experiments with B16F10 melanoma grafted mice models. **(A)** Experimental schedule. Starting at 7 days post implantation, mice were intravenously treated with each phage and/or GM-CSF once per day for 10 days. **(B)** Tumor mass grown on the right flank of mice. **(C)** Isolated tumor mass after mice sacrifice. WT T7, wild type phage T7; T7-pep42, phage T7 displaying pep42 and T7-pep42_G, phage T7 displaying pep42 and expressing GM-CSF; T7-pep42 + G, phage T7 displaying pep42 and externally added protein cytokine GM-CSF; +G, externally added protein cytokine GM-CSF. **(D)** Tumor growth from 7 days post implantation till 16 days p.i. in each treatment. **(E)** Measurement of tumor volume after mice sacrifice of each treatment (*n* = 5/group). For statistical analysis, one way ANOVA was performed and Tukey’s test was conducted; ^*^ or ^**^above each vertical bar indicates statistical significance of each test to the control; ^*^ or ^**^above each horizontal bar indicates statistical significance of each test between corresponding pairs. **(F)** Survival of mice bearing tumor mass was monitored for each treatment for 25 days post implantation (*n* = 5/group). Log rank test was used to test the significance.

**Figure 4:**
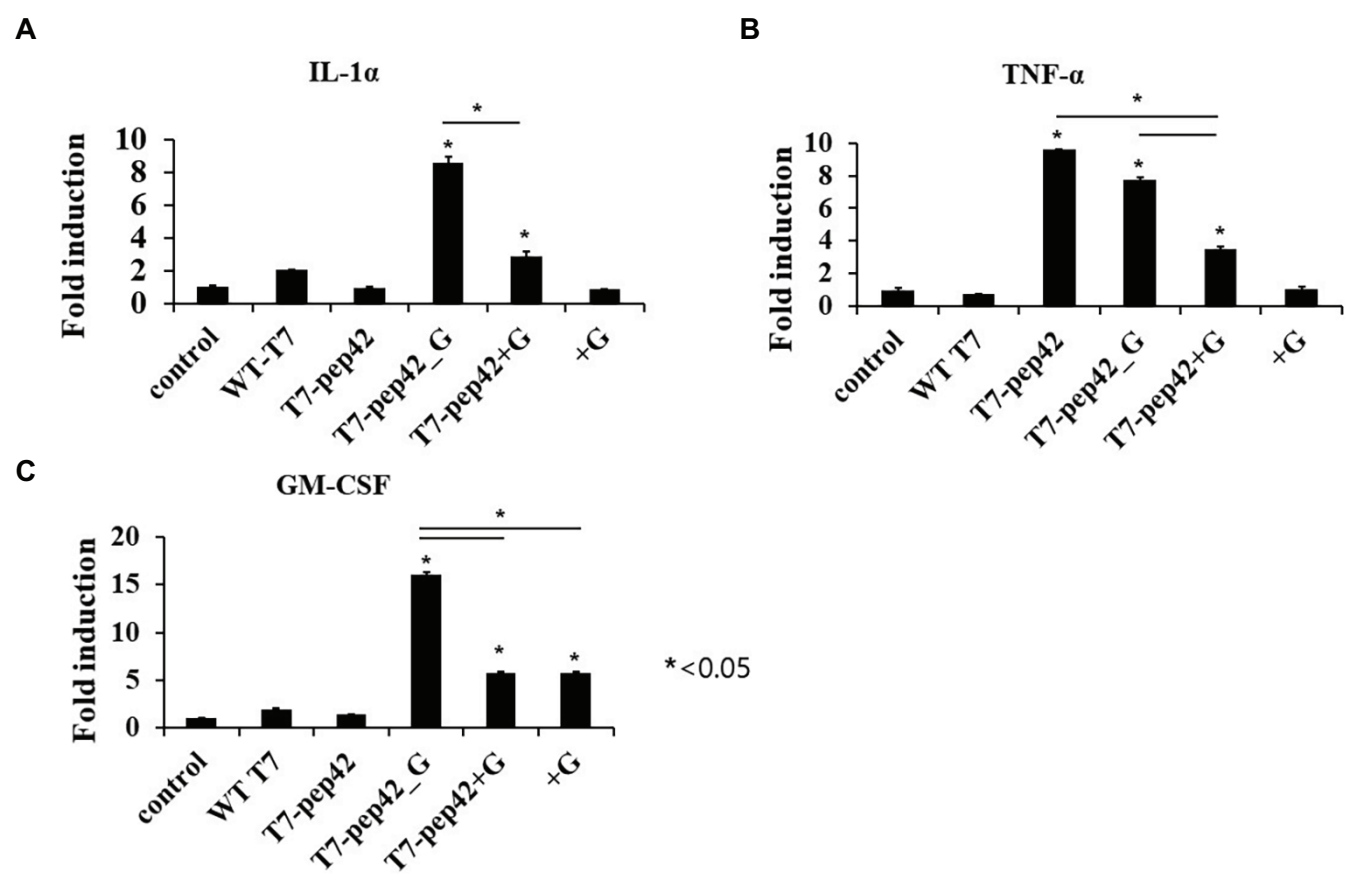
Measurement of serum cytokine levels of tumor bearing mice in each treatment. Mice were sacrificed after 10 daily treatments of each phage and/or GM-CSF, and serum cytokine was measured using cytokine ELISA (*n* = 3). **(A)** IL1-α. **(B)** TNF-α. **(C)** GM-CSF. WT T7, wild type phage T7; T7-pep42, phage T7 displaying pep42; T7-pep42_G, phage T7 displaying pep42 and expressing GM-CSF; T7-pep42 + G, phage T7 displaying pep42 and externally added protein cytokine GM-CSF; +G, externally added protein cytokine GM-CSF. For statistical analysis, one way ANOVA was performed and Tukey’s test was conducted. ^*^Above each vertical bar indicates statistical significance of each test to the control. ^*^Above each horizontal bar indicates statistical significance of each test between corresponding pairs.

Since the engineered phage T7 was seen to home in tumor mass *in vivo* and to express GM-CSF *in vitro*, we next explored whether this phage could inhibit tumor growth *in vivo*. *In vitro* cultured B16F10 murine melanoma cells were implanted into mice and allowed to grow for 7 days before the start of phage treatment. Phages were intravenously injected into mice once every day for 10 days and the survival, and changes in, tumor masses were observed (Figures 3A,B). Tumor mass was measured 7, 10, 13, and 16 days post implantation with various treatments (Figures 3C–E). Phage T7 displaying homing peptide and expressing GM-CSF (T7-pep42_G) inhibited tumor growth by 72% compared to the untreated control. Treatment with phage T7 displaying pep42 (T7-pep42) or in combination with externally added protein GM-CSF led to a decrease in tumor volume by 50%. No additional effects were observed at the given concentration of GM-CSF. The amount of total GM-CSF available in the body could be one determining factor for the efficacy of this treatment (see Figure 4). Alternatively, the availability of GM-CSF in the tumors’ microenvironment could be another determinant. Isolated tumor mass after sacrifice is shown for each treatment (Figure 3C). Forty percent of untreated mice survived, while 100% of mice treated with phage T7 displaying the homing peptide and expressing GM-CSF (T7-pep42_G) survived at the end of the experiment (Figure 3F). Untreated mice began to die from day 5 and 60% died by day 15. Sixty percent of mice treated with wild type T7 displaying homing peptide (T7-pep42) or with protein GM-CSF survived, but protein GM-CSF-treated mice had earlier deaths. Eighty percent of wild type T7 or T7 displaying pep42 plus added GM-CSF survived, but wild type T7-treated mice had earlier deaths. One can note the following, in Figure 2, the live imaging was performed at 7 days post graft and hours after IV injection of phages. Thus, the tumor sizes were identical at that particular time point. In Figure 3, tumor mass was measured from 7 to 16 days post graft, where effects of injected phages were observed. Thus the initial tumor sizes between the two experiments were different.

Animal cells are not natural hosts for bacteriophages and we cannot expect cell lysis from phage multiplication. One possibility for the lysis of tumor cells is immunological attack. Since the recruitment of immune cells to the tumor could be mediated by various cytokines, we next checked the increase in serum cytokine after the administration of the phages (Figure 4). Three inflammatory cytokines, IL-1α, TNF-α, and GM-CSF, were measured in mice serum after each treatment. Eight- or three-fold increases in serum IL-1α were observed when the mice were administered with phage T7 displaying pep42 and expressing GM-CSF (T7-pep42_G) or phage T7 displaying pep42 plus the externally added protein GM-CSF (T7-pep42 + G), respectively. Neither the phage alone nor the protein GM-CSF alone lead to any increases (Figure 4A). However, homing phage T7 displaying pep42 (T7-pep42) alone could strongly increase serum TNF-α (Figure 4B). The latter effect decreased in the presence of GM-CSF. T7-pep42 alone induced TNF-α, but not other cytokines. In case of viral infection, inflammatory cytokines are induced at different levels. For example, Epstein-Barr virus infection in B or T cells induced TNF-α, but not IL-1α (Lay et al., 1997). In another example, SARS-CoV2 infection led to a preferential production of TNF-α, resulting in loss of germinal center cells (Kaneko et al., 2020). Phage T7 may preferentially induce TNF-α rather than other cytokines. Phage T7 displaying pep42 and expressing GM-CSF (T7-pep42_G) induced the highest level of the cytokines (Figure 4C). T7 displaying the homing peptide (T7-pep42) alone did not induce the production of GM-CSF. Taken together, both T7 displaying the homing peptide and GM-CSF were the determinants for the induction of IL1-α, while the presence of T7 displaying the homing peptide is the major determinant for the induction of TNF-α. T7 displaying pep42 (T7-pep42) was not an inducer of GM-CSF in these mice.

**Figure 5:**
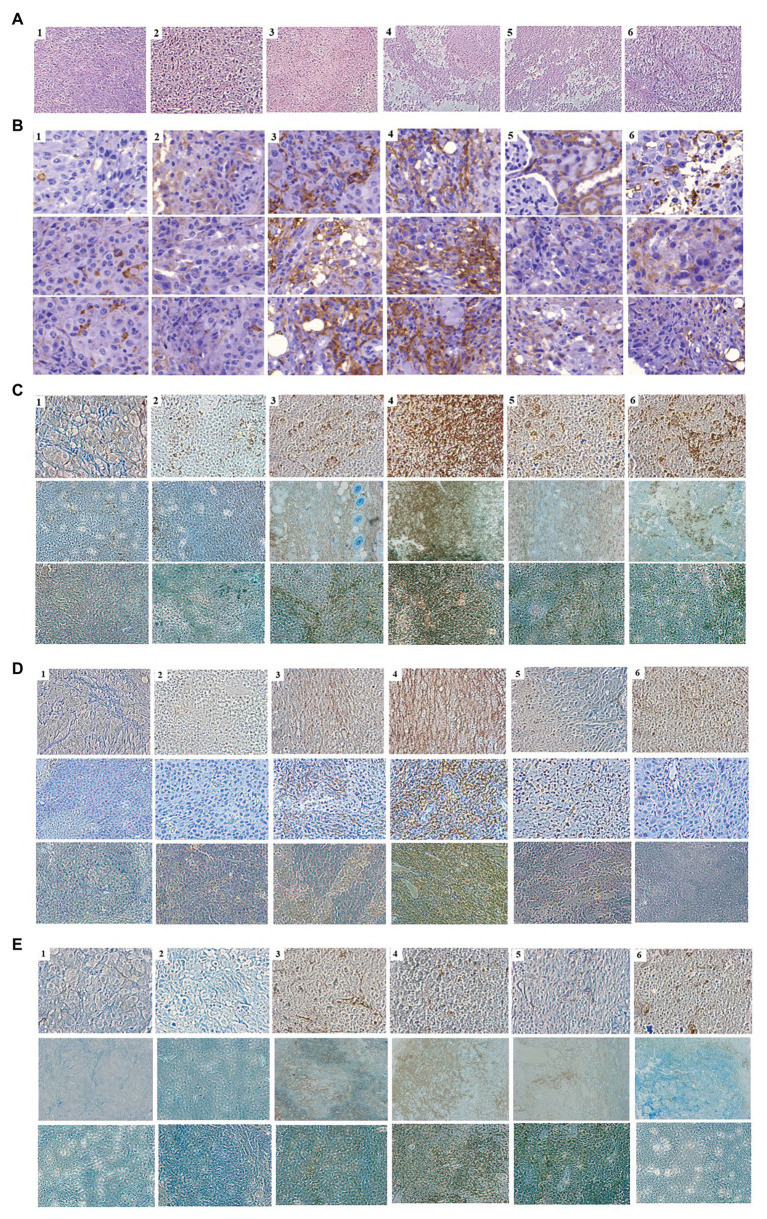
Immunohistochemical analysis of immune cells in tumor tissue from mice treated with various phages and/or GM-CSF. 1, control; 2, WT T7; 3, T7-pep42; 4, T7-pep42_G; 5, T7-pep42 + G; 6, +G. **(A)** H&E staining of tumor tissue for each treatment. Massive necrotic or damaged tumor cells are seen after treatment with recombinant phages. **(B)** Infiltration of macrophages. **(C)** Infiltration of CD11 positive dendritic cells. **(D)** Infiltration of CD8 positive T cells. **(E)** Infiltration of CD49 positive natural killer (NK) cells. Infiltrated cells are antibody-stained and shown in dark brown. For, **(B–E)**, multiple sections of tumor tissue were stained immunohistochemically and observed for each immune cell.

As the expression of cytokines could lead to the activation and recruitment of immune cells to tumor mass, mice bearing tumor mass were treated with phages and/or cytokine GM-CSF, and immunohistochemical observation was performed (Figure 5). Massive necrotic or damaged tumor cells were seen after treatment with the recombinant phage (Figure 5A). Tumor destruction and the shrinkage of cells were most prominent in mice treated with phage T7 displaying pep42 and GM-CSF, which was either expressed from the phage or externally added. Wild type T7, T7 displaying pep42, or externally added protein GM-CSF alone, induced a limited destruction of tumor mass and shrinkage of cells. The highest degree of macrophage infiltration was observed when both T7 displaying pep42 and GM-CSF were present (Figure 5B). Lesser infiltration was seen when T7 displaying pep42 or GM-CSF alone was treated. For DC or cytotoxic T cells, T7 displaying pep42 and expressing GM-CSF strongly recruited the immune cells (Figures 5C,D). Considering the amount of total GM-CSF detected (Figure 4C), GM-CSF seems to play a significant role in recruiting the immune cells. For natural killer (NK) cells, little recruitment was seen with any treatment (Figure 5E). Multiple sections of tumor tissue were stained immunohistochemically and observed for each immune cell.

**Figure 6:**
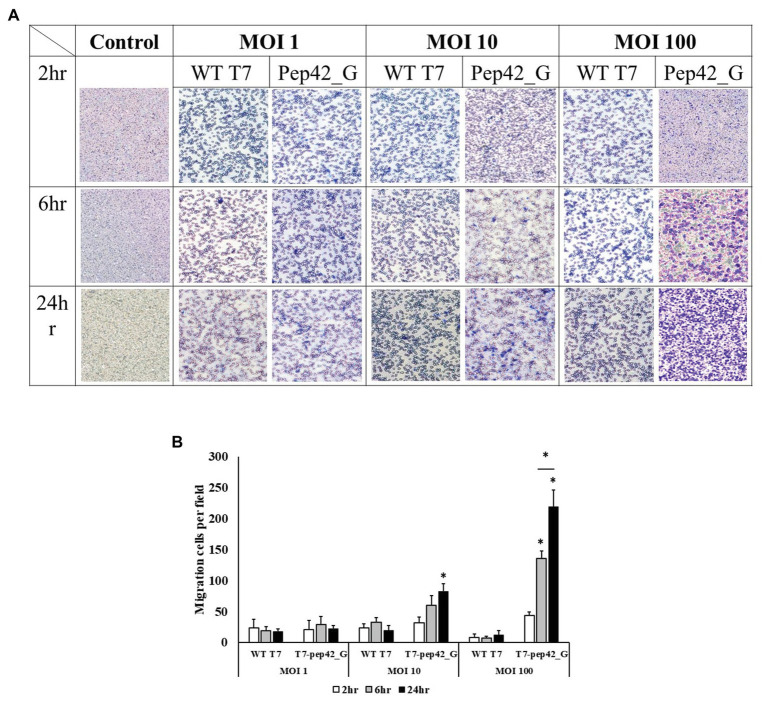
Transwell migration assay of macrophages. Cultured B16F10 cells in the lower chamber were treated with three different titers (MOI 1, 10, or 100) of wild type T7 (WT T7) or T7 displaying the homing peptide and harboring an mammalian expression cassette of GM-CSF (T7-pep42_G). Macrophages (RAW264.7) in the upper chamber were allowed to migrate for three different time periods. **(A)** Staining and visualization of membrane after migration. White pores are seen from the membrane and macrophages are stained with crystal violet. **(B)** Three random fields were chosen and the migrated cells were quantitated. For statistical analysis Tukey’s test was performed. ^*^Above each vertical bar indicates statistical significance of each test to the control. ^*^Above each horizontal bar indicates statistical significance of each test between corresponding pairs.

To observe phage-induced macrophage infiltration quantitatively *in vitro*, we performed a transwell migration assay. Various doses of either wild type T7 or T7 displaying pep42 and expressing GM-CSF (T7-pep42_G) were added and macrophage migration was detected at various points in time (Figure 6). It was seen that wild type T7 hardly induced any migration of macrophages, while T7 expressing GM-CSF induced a massive recruitment of macrophages in both dose- and time-dependent manners.

## Discussion

In this experiment, a very efficient homing of phage T7 displaying pep42, targeting cell surface receptor grp78, was verified both *in vitro* and *in vivo*. In addition, *in vivo* imaging showed that accumulation of wild type T7 (without tropism for B16F10 cells) occurred at the tumor, although at a much lesser degree. Earlier literature reported the accumulation of phages in cancer tissue and the inhibition of tumor growth in 1940 (Budynek et al., 2010). This observed accumulation was both intratumoral and peritumoral. We speculate that the peritumoral accumulation of wild type phages in the latter experiment was in the tumor vasculature (Forbes et al., 2003). For example, bacteria were also shown to accumulate in the chaotic vasculature of tumors. Bacteria are better at infiltrating inside tumors thanks to their motility, which phages do not have (Forbes, 2010). Nonetheless, a small portion of phages were seen inside tumor masses *in vivo* in this experiment. An earlier experiment using the bacteriophage lambda also reported that unmodified phage particles were capable of transducing mammalian cells *in vivo*, although much less efficiently than the surface modified phage targeting mammalian cells (Lankes et al., 2007).

Once attached to the cell surface, phage T7 particle was efficiently internalized and phage genomic DNA was delivered to nuclei followed by the expression of gene encoding GM-CSF from the cassette. It is coherent with previously reported gene deliveries using native phage lambda (Lankes et al., 2007), phage lambda displaying cyclizable RGD peptide to COS-1 cells (Dunn, 1996), phage M13 displaying epidermal growth factor (EGF) to COS-1 cells (Kassner et al., 1999), M13 targeting Her-2 receptor (Urbanelli et al., 2001), or AAVP displaying RGD peptide (Hajitou et al., 2006; Trepel et al., 2009; Kia et al., 2012; Stoneham et al., 2012; Smith et al., 2017; Przystal et al., 2019). Trafficking of internalized phage particles was shown to be mediated by clathrin-mediated endocytosis followed by endo-lysosomal delivery (Stoneham et al., 2012). The latter is a common pathway for internalized materials and escaping from the endo-lysosome seems to be critical for more efficient expression of phage transgenes.

Increases in serum cytokines may be a factor leading to tumor regression in this experiment. Recently approved oncolytic virus T-Vec harbors two copies of gene encoding GM-CSF (Conry et al., 2018). GM-CSF promotes DC accumulation at sites of inflammation and enhances antigen presenting cell functions (Kaufman et al., 2014). Since GM-CSF was expressed from transduced tumor cells, the local concentration is expected to be higher than in total serum. Two other proinflammatory cytokines, IL-1α and TNF-α, were also increased in the presence of T7 displaying homing peptide and harboring expression cassette of GM-CSF. In addition, TNF-α increased in the presence of T7 displaying the homing peptide without expression of GM-CSF, suggesting that locally accumulated, high titer of the phages themselves could stimulate cytokine production. Interestingly, less increases of TNF-α were observed, where GM-CSF was expressed. Therefore, there may be interference between the productions of the two cytokines in this experiment.

Changes in the tumor microenvironment, including locally accumulated phages and increases in proinflammatory cytokines accompanied by the recruitment of various immune cells, seems responsible for tumor regression. In fact, macrophages were found in tumor tissue when various phages and/or GM-CSF were administered. On the other hand, DC and CD8+ T cells were predominantly found in tumor tissue when the T7 displaying the homing peptide and harboring expression cassette of GM-CSF was administered. Local expression of GM-CSF might lead to enhanced T-cell priming by DC. Antigen priming by DCs and the direct killing of tumor cells by T cells are the most powerful way of bringing about immune-mediated tumor regression. We could observe the same set of immune cells clearly infiltrating tumor tissue, where homing phages accumulated and GM-CSF was produced by the tumor cells themselves, which could lead to locally increased concentrations. In case of NK cells, we could not observe any strong recruitment. One study reported a dual role of GM-CSF in recruiting NK cells (Nandagopal et al., 2015). At high concentrations, GM-CSF strongly induced migration of NK cells, while at low concentrations, it induced hyper-polarization, immediate arrest of NK cells, and little/or no NK-cell migrations. The observation that macrophage, one of the immune cells recruited *in vivo*, also migrated to tumor cells treated with phage T7 displaying the homing peptide and harboring expression cassette of GM-CSF in time- and dose-dependent manners in an *in vitro* transwell assay is coherent with *in vivo* results.

The immuno-modulatory nature present in the mucosal immunity of bacteriophages was recently reported (Gogokhia et al., 2019). The authors described that bacteriophages or phage DNA could stimulate IFN-γ production *via* TLR-9 sensing, resulting in exacerbated colitis. A greater and greater body of evidence suggests relationships between phages and mammalian immunity. There have been several reports describing tumor regression in the presence of phages. In this experiment, we have demonstrated advancements in the utilizing of phages for this purpose. First, by displaying that the homing peptide (pep42) is more highly selective for target cells when compared to KGD or RGD peptides previously described (Dabrowska et al., 2004; Quinn et al., 2017). Although a report had already described the peritumoral injection of phage M13 displaying a homing peptide (Eriksson et al., 2007), we utilized intravenous injections and obtained greater tumor regression, suggesting better targeting methods and the increased stability of our system. T7 has much more packaging capacity when compared to M13, and the double stranded nature of genomic DNA has a better expression and prolonged stability inside mammalian cells. Unlike AAVP (Hajitou et al., 2006), T7 has no DNA of animal virus origin, leading to less possible side effects. Taken together, the combination of tumor-targeting bacteriophages and intratumoral expression of GM-CSF from transduced phage DNA can efficiently stimulate the immune system, leading to tumor regression.

## Data Availability Statement

The datasets generated for this study are available on request to the corresponding author.

## Ethics Statement

The animal study was reviewed and approved by Ethical Committee for Animal Experiments of Hankuk University of Foreign Studies.

## Author Contributions

HM conceived and designed the experiments. YH performed the experiments and generated the data. HM wrote the manuscript. All authors contributed to the article and approved the submitted version.

### Conflict of Interest

The authors declare that the research was conducted in the absence of any commercial or financial relationships that could be construed as a potential conflict of interest.
